# Outcomes of total hip and knee arthroplasty in special populations: a synopsis and critical appraisal of systematic reviews

**DOI:** 10.1186/s42836-023-00190-7

**Published:** 2023-07-06

**Authors:** Dimitris Challoumas, David Munn, Henrietta Stockdale, Nigel Ng, Michael McCormick, Tareq Altell, Shaheer Joiya, James Horton, Bryn Jones

**Affiliations:** Department of Trauma and Orthopaedic Surgery, Glasgow Royal Infirmary, 84 Castle Street, Glasgow, G4 0SF Scotland UK

**Keywords:** Total hip arthroplasty, Total knee arthroplasty, Complication, Outcome, Risk factor, Systematic review

## Abstract

**Introduction:**

This study aimed to present and critically appraise the best available evidence investigating associations between some pre-defined patient-related characteristics and perioperative complications or other outcomes in THA and TKA.

**Methods:**

Electronic databases were searched (Medline, EMBASE, Scopus, CENTRAL) for systematic reviews assessing the following pre-defined patient-related characteristics as possible risk factors for worse peri-operative outcomes in THA and TKA: smoking, alcohol excess, rheumatoid arthritis, human immunodeficiency virus infection, hepatitis C virus infection, mental health conditions, and solid organ transplantation. Our primary outcome was periprosthetic joint infection. Results were analysed separately for THA, TKA and THA/TKA (mixed data).

**Results:**

Based on at least two systematic reviews being in agreement, the following patient-related characteristics were associated with increased incidence of complications as follows: a) Smoking for all-cause revision in THA, for periprosthetic joint infection in TKA and THA/TKA; b) alcohol excess for periprosthetic joint infection in THA/TKA; c) human immunodeficiency virus for periprosthetic joint infection in THA/TKA; d) hepatitis C virus for overall complications, periprosthetic joint infection and all-cause revision in THA and THA/TKA, and for overall complications in TKA. Our study found conflicting evidence for a) smoking as a risk factor for periprosthetic joint infection and aseptic loosening in THA; b) human immunodeficiency virus as a risk factor for all-cause revision for THA/TKA; c) hepatitis C virus as a risk factor for periprosthetic joint infection and all-cause revision in TKA. No certainty of evidence was assigned to these results as this was not assessed by the authors of the majority of the included systematic reviews.

**Conclusion:**

We found that smoking, excess alcohol consumption, RA, and infection with HIV and HCV were associated with a higher incidence of periprosthetic joint infection in one or both of THA and TKA or mixed THA/TKA data. All our results should be interpreted and communicated to patients with caution as the quality of the included systematic reviews was generally poor.

## Introduction

Total hip arthroplasty (THA) and total knee arthroplasty (TKA) are amongst the commonest orthopaedic procedures generally leading to a substantial improvement in pain, function and quality of life [1–4]. Despite continuous attempts for optimal surgical techniques and implants over the last decades, a small proportion of patients experience complications, which in rare cases are life-changing or even life-threatening [5].

Appropriate patient selection is perhaps the most important step a surgeon can take to minimise complications of a THA or TKA. Patient-related characteristics have received a lot of attention in research over the last years, and some surgeons may even refuse to operate on specific patients because of the presence of related risk factors [2, 6, 7]. Patient-related characteristics may be modifiable (e.g. obesity, smoking and alcohol consumption) or non-modifiable (e.g. previous septic arthritis, diabetes mellitus and age) and whilst some of them, such as obesity and diabetes, are confirmed risk factors for complications, some others are controversial due to inadequate or conflicting evidence [8–11]. Although single studies including large populations can be informative, systematic reviews (SRs) combining the results of similar studies have the highest level of evidence and should be preferentially considered for clinical decisions and policy making [12].

This study aimed to systematically review the literature on some of the patient-related characteristics that are not universally accepted as risk factors of complications after primary THA and TKA. We expect that presentation of the relevant literature will help both implicated healthcare professionals and patients alike consider complication risks when making decisions about a THA or TKA.

## Materials and methods

### Search strategy

**Fig. 1:**
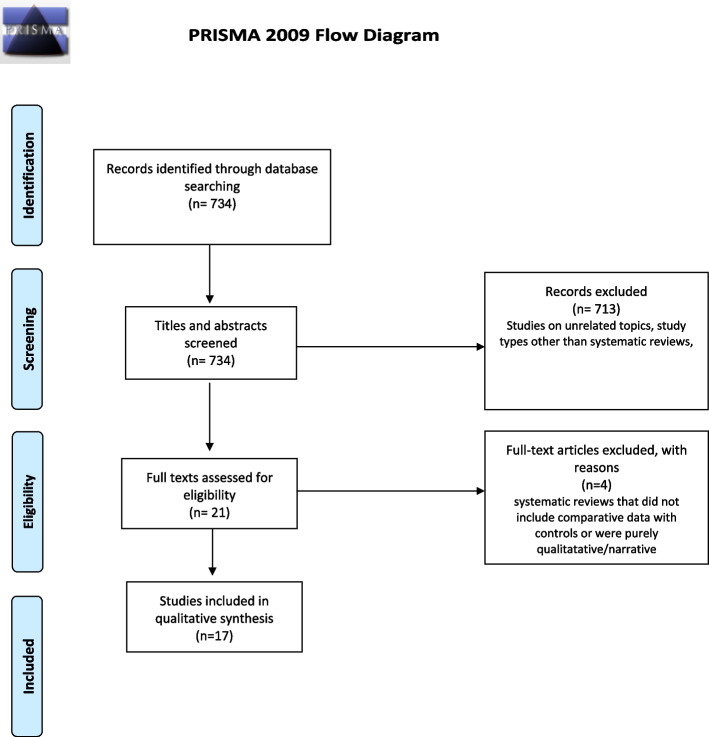
PRISMA diagram showing the article screening process

Studies were identified through a literature search through Medline, Ovid, EMBASE and Cochrane databases from inception to May 2022 (Fig. 1). Combinations of the following keywords were used in “all fields”: “joint arthroplasty”, “joint replacement”, “knee arthroplasty”, “knee replacement”, “hip arthroplasty”, “hip replacement”, “hepatitis”, “HIV”, “human immunodeficiency virus”, “smoking”, “tobacco”, “alcohol”, “psychiatry*”, “substance”, “mental health”, “depression”, “schizophrenia”, “psychosis”, “bipolar”, “inflammatory arthr*”, “rheumatoid”, “crystal arthr*”, “*gout”, “seronegative arthr*”, “haemophilia”, “hemophilia”, “haemochromatosis”, “hemochromatosis”, “*organ transplant”, “kidney transplant”, “liver transplant”, “lung transplant”, “heart transplant”, “peripheral vascular disease”, “post-traumatic”, “septic”, “infection”.

### Eligibility criteria

Eligible studies had to 1) be SRs and compare the patient-related characteristic of interest with a control population (patients without the characteristic of interest or “general population”), 2) include peri-operative complications or other post-operative outcomes of THA or TKA in their outcome, and 3) report results of statistical tests (odds ratio (OR); relative risk (RR); hazard ratio (HR); mean difference (MD)). We excluded 1) studies other than SRs (including narrative reviews), 2) studies published in languages other than English, 3) those including revision arthroplasty or partial knee arthroplasty, and 4) those where the arthroplasty was performed for the treatment of a fracture. No time criteria were applied.

The patient-related characteristics or factors of interest were pre-defined following discussion and consensus agreement among the first and last authors and included: infection with human immunodeficiency virus (HIV) or hepatitis C virus (HCV), alcohol excess, use of tobacco, mental health conditions, haemophilia, haemochromatosis, post-traumatic arthritis, post-septic arthritis, inflammatory or crystal arthritis (including rheumatoid arthritis; RA), peripheral vascular disease, and solid organ transplantats.

### Patients, interventions, comparators and outcomes

We compared patients with versus without the characteristics of interests (potential risk factors for worse outcomes) for peri-operative complications and other post-operative outcomes measured at all follow-up time points. Our primary outcome was periprosthetic joint infection (PJI). Secondary outcomes included a) overall complications, b) overall surgical complications, c) overall medical complications, d) aseptic loosening, e) dislocations (THA only), f) revision, g) length of stay (LOS), h) hospital re-admission, and i) mortality.

### Quality assessment

The AMSTAR-2 tool was used to critically appraise and rate the overall confidence in the results of each included SR [13]. The overall confidence was “high” (no or one non-critical weakness, no critical flaws), “moderate” (> 1 non-critical weakness, no critical flaws), “low” (one critical flaw with or without non-critical weaknesses), or “critically low” (more than one critical flaw with or without non-critical weaknesses).

### Data handling—synthesis

Data of interest regarding the characteristics of the included SRs and their findings were extracted by two authors independently and were tabulated in Microsoft Word. These were categorised based on patient-related characteristics of interest. Overall results were obtained after qualitative pooling based on direction of effect (i.e., statistically higher, ↑; lower, ↓; and no difference, ↔) for THA, TKA and THA/TKA (mixed data) separately.

Where “infection” was reported as a general outcome in the context of implant-related complications, this was assumed to be the same as PJI. When the timing of outcomes was not specifically reported (i.e., 90-day re-admissions), the results were described for maximum follow-up of each study within that SR.

### Statistical analysis

No statistical tests or analyses were performed by the authors of the present review. Differences were stated to be statistically significant (or just “significant”, or “significantly higher and lower”, etc.) where statistical tests in the included SRs returned a *P* < 0.05. Where data of two or more SRs assessing the same patient-related characteristic with the same type of arthroplasty (THA, TKA or mixed THA/TKA data) were pooled, the overall result was based on direction of effect only without accompanying numerical values. Possible overall results included a) statistically higher incidence (↑), b) statistically lower incidence (↓), and c) no difference ( ↔) when the results of the combined SRs were generally consistent, or d) “conflicting evidence” when they were inconsistent. I^2^ < 50% was interpreted as low statistical heterogeneity and I^2^ ≥ 50% as significant statistical heterogeneity.

## Results

### Characteristics of included systematic reviews

A total of 17 SRs were included [1, 2, 6, 7, 14–26] (Table 1); these investigated THA and/or TKA outcomes and complications in the following patient-related characteristics: a) Smoking (9 SRs), alcohol excess (4 SRs), RA (3 SRs), Hepatitis C (2 SRs), HIV (two SRs), depression (1 SR), solid organ transplants (1 SR). Four SRs assessed more than one characteristic [2, 6, 7, 14]. The total number of studies in SRs ranged from 6–66 and all these were prospective cohort or retrospective case–control studies. The included SRs assessed outcomes in THA alone (5 SRs), TKA alone (2 SRs), both THA and TKA (4 SRs), and mixed THA/TKA outcomes (8 SRs). The Newcastle–Ottawa scale was used in 10 SRs (59%) to assess study quality. OR, RR, and HR were used for statistical analysis in 9, 7, and 2 SRs, respectively. Mean follow-up in the studies within the included SRs ranged from immediate postoperative to 26 years.

**Table 1:** Main characteristics of the 17 included systematic reviews

**SR**	**Journal**	**Characteristic**	**Arthroplasty type**	**Total number of studies**	**Mean follow up**	**Risk of bias/Quality assessment tool**	**Strength of evidence assessed?**	**Outcomes meta-analysed**	**Meta-analysis statistic**	**Confidence in results as per AMSTAR-2**
Singh (2011) [1]	*J Rheumatol*	Tobacco use	THA	21	Immediate post-op–8 y	Newcastle–Ottawa Scale	No	Overall complications, revision, aseptic loosening	RR	Critically low
Ravi et al. (2012) [19]	*Arthritis Rheum*	RA	THA, TKA	40	Not reported	Yes (arbitrary method)	Yes (arbitrary method)	Revision, infection, hip dislocation, 90-day mortality, venous thromboembolism	OR	Critically low
Teng et al. (2015) [17]	*PLoS One*	Tobacco use	THA	6	2 m–13 y	Newcastle–Ottawa Scale	No	Aseptic loosening, deep infection, all-cause revision, dislocation, LOS	RR	Critically low
Kunutsor et al. (2016) [2]	*PLoS One*	Tobacco use, Alcohol excess (among others)	THA/TKA	66	1–17 y	Newcastle–Ottawa Scale	No	PJI	RR	Critically low
Dimitriou et al. (2017) [22]	*JBJS Rev*	HIV	THA/TKA	21	Not reported	Not performed	No	Overall complications, PJI, implant survivorship	RR	Critically low
Kong et al. (2017) [6]	*Int Wound J*	Tobacco use, Alcohol excess, RA (among others)	THA/TKA	24	Not reported	Newcastle–Ottawa Scale	No	PJI	OR	Critically low
Lee et al. (2017) [20]	*Knee Surg Sports Traumatol Arthrosc*	RA	TKA	13	Not reported	Newcastle–Ottawa Scale	No	Superficial infection, deep infection, revision for infective and non-infective causes	OR	Critically low
Bedard et al. (2019) [15]	*J Arthroplasy*	Tobacco use	THA/TKA	14	30 d-5 y minimum	Newcastle–Ottawa Scale	No	PJI	OR	Critically low
O’Neill et al. (2019) [21]	*Orthop Rev (Pavia)*	HIV	THA/TKA	19	Not reported	Newcastle–Ottawa Scale	No	Infection, all-cause revision	RR	Critically low
Wei et al. (2019) [23]	*J Orthop Sci*	Hepatitis C	THA, TKA, THA/TKA	10	Max 117 months	Downs and Black tool	No	Overall complications, infection, revision, LOS	HR	Critically low
Bojan et al. (2020) [16]	*Arthroplast Today*	Tobacco use	THA	10	1 m–5 y	Newcastle–Ottawa Scale	No	Superficial infection, deep infection, revision due to infection	OR	Critically low
Kim & Kim 2021 [25]	*J Arthroplasy*	Depression	TJA, THA, TKA	9	Not reported	MINORS	No	30-day and 90-day re-admission rates	OR	Critically low
Ren et al. (2021) [7]	*BMC Musculoskelet Disord*	Tobacco use, Alcohol excess, RA (among others)	THA	40	2.92 y	Newcastle–Ottawa Scale	No	PJI	RR	Critically low
Resende et al. (2021) [14]	*Knee Surg Sports Traumatol Arthrosc*	Tobacco use, Alcohol excess (among others)	THA/TKA	37	30 d–11.2 y	ROBBINS-I	Yes (GRADE)	PJI	OR	Critically low
Cheng et al. (2022) [24]	*Orthop Traumatol Surg Res*	Hepatitis C	THA, TKA, THA/TKA	15	Immediate post-op–10 y	Downs and Black tool	No	Overall complications, surgical complications, medical complications, overall infection, PJI, blood transfusion, revision, LOS	OR	Critically low
He et al. (2022) [18]	*Bosn J Basic Med Sci*	Tobacco use	TKA	13	2–26 y	Newcastle–Ottawa Scale	No	TKA incidence, overall complications, surgical complications (superficial infection, deep infection, wound dehiscence, revision), medical complications (deep vein thrombosis, pulmonary embolism, blood transfusion, urinary tract infection, pneumonia) mortality	RR, HR	Low
Kim et al. (2022) [26]	*Hip & Pelvis*	Solid organ transplant	THA	10	> 2 y	MINORS	No	Medical complications (cardiac, DVT, PE, AKI, pneumonia), surgical complications (transfusion, dislocation, PJI, aseptic loosening, revision), readmission, 90-day mortality	OR	Critically low

*d* Days, *HIV* Human immunodeficiency virus, *HR* Hazard ratio, *LOS* Length of stay, *m* months, *OR* Odds ratio, *PJI* Periprosthetic joint infection, *RA* Rheumatoid arthritis, *RR* Relative risk (risk ratio), *THA* Total hip replacement, *TKA* Total knee replacement, *y* Years

### Quality assessment results

We found that all studies had more than one critical flaw. Even when the seven AMSTAR critical domains as described by the creators of the tool were considered on their own, 16 out of 17 SRs had critical flaws in at least one of these seven items [13]. Only one SR [18] fulfilled all seven critical domains, and therefore the confidence in their results was rated as “low”. That of the other 16 SRs was rated as “critically low” (Table 2).

**Table 2:** Critical appraisal outcomes assessed using the AMSTAR-2 tool for the 17 included systematic reviews. Each numbered column represents one of the 16 items of the tool. Column numbers in bold illustrate the 7 AMSTAR critical domains

	1	2	3	4	5	6	7	8	9	10	11	12	13	14	15	16
Singh (2011) [1]	N	N	N	PY	Y	N	Y	PY	Y	N	N	N	N	N	N	Y
Ravi et al. (2012) [19]	Y	PY	N	PY	Y	Y	N	N	PY	N	Y	N	N	N	Y	N
Teng et al. (2015) [17]	N	N	N	PY	Y	Y	N	PY	Y	N	Y	N	N	N	Y	Y
Kunutsor et al. (2016) [2]	N	Y	N	PY	N	Y	N	PY	Y	N	Y	N	N	Y	Y	Y
Dimitriou et al. (2017) [22]	N	N	N	PY	N	N	N	N	N	N	N	N	N	N	N	N
Kong et al. (2017) [6]	N	N	N	PY	Y	Y	N	N	Y	N	N	N	N	N	Y	N
Lee et al. (2017) [20]	N	N	N	PY	Y	Y	N	N	Y	N	N	N	N	N	N	Y
Bedard et al. (2019) [15]	N	N	N	PY	Y	N	N	PY	Y	N	N	N	N	N	Y	Y
O’Neill et al. (2019) [21]	N	N	N	PY	Y	N	N	PY	Y	N	Y	N	N	Y	N	Y
Wei et al. (2019) [23]	N	N	N	PY	Y	Y	N	PY	Y	N	Y	N	N	N	Y	Y
Bojan et al. (2020) [16]	N	N	N	PY	Y	N	N	PY	Y	N	Y	N	N	N	Y	Y
Kim & Kim 2021 [25]	N	N	N	PY	Y	Y	N	PY	Y	N	N	N	N	N	Y	Y
Ren et al. (2021) [7]	N	N	N	PY	Y	N	N	PY	Y	N	Y	N	Y	Y	Y	Y
Resende et al. (2021) [14]	N	Y	N	PY	Y	Y	N	PY	Y	N	Y	Y	Y	Y	Y	Y
Cheng et al. (2022) [24]	Y	N	Y	PY	N	N	N	PY	Y	N	N	N	N	N	Y	Y
He et al. (2022) [18]	N	Y	N	PY	Y	Y	Y	PY	Y	N	Y	Y	Y	Y	Y	Y
Kim et al. (2022) [26]	Y	N	Y	PY	Y	N	N	PY	Y	N	Y	N	N	N	Y	Y

*Y* Yes, *PY* Partial yes, *N* No

### Findings of review

Table 3 summarises the main results of the included SRs which are grouped based on the patient-related characteristic assessed and the overall results as pooled qualitatively by the authors of the present review. The individual sections below summarise the results of our qualitative pooling based on direction of effect for each outcome measure as shown in Table 3.

- Tobacco use:
  - ◦ THA: Increased incidence of overall complications (1 SR), all-cause revision (2 SRs) and mortality (1 SR); no difference in incidence of dislocations (1 SR) and LOS (1 SR); conflicting evidence for PJI (4 SRs) and aseptic loosening (2 SRs).
  - ◦ TKA: Increased incidence of overall complications (1 SR), PJI (2 SRs), overall surgical complications (1 SR) and all-cause revision (1 SR); no difference in incidence of overall medical complications (1 SR) and mortality (1 SR).
  - ◦ THA/TKA (mixed data): Increased incidence of PJI (4 SRs).
- Alcohol excess:
  - ◦ THA: Increased incidence of PJI (1 SR).
  - ◦ THA/TKA (mixed data): Increased incidence of PJI (3 SRs).
- Rheumatoid arthritis:
  - ◦ THA: Increased incidence of PJI (3 SRs) and dislocations (1 SR); no difference in mortality (1 SR) or all-cause revision incidence (2 SRs).
  - ◦ TKA: Increased incidence of PJI (1 SR); no difference in mortality (1 SR) or all-cause revision incidence (1 SR).
  - ◦ THA/TKA (mixed data): Increased incidence of PJI (1 SR).
- Human immunodeficiency virus infection:
  - ◦ THA/TKA (mixed data): Increased incidence of PJI (2 SRs) and overall complications (1 SR); conflicting evidence for all-cause revision (2 SRs).
- Hepatitis C infection:
  - ◦ THA: Increased incidence of overall complications (2 SRs), overall surgical complications (1 SR), overall medical complications (1 SR), PJI (2 SRs), all-cause revision (2 SRs), LOS (1 SR).
  - ◦ TKA: Increased incidence of overall complications (2 SRs), overall surgical complications (1 SR), overall medical complications (1 SR), and LOS (1 SR); conflicting evidence for PJI (2 SRs) and all-cause revision (2 SRs).
  - ◦ THA/TKA (mixed data): Increased incidence of overall complications (2 SRs), overall surgical complications (1 SR), overall medical complications (1 SR), PJI (2 SRs), all-cause revision (2 SRs), LOS (1 SR).
- Mental health conditions (depression):
  - ◦ THA: No difference in 90-day re-admission rates (1 SR).
  - ◦ TKA: Increased incidence of 90-day re-admission (1 SR).
  - ◦ THA/TKA (mixed data): Increased incidence of 90-day re-admission (1 SR).
- Solid organ transplant:
  - ◦ THA: No difference in PJI (1 SR), dislocation (1 SR), and aseptic loosening rates (1 SR). Increased incidence of 90-day mortality (1 SR) and re-admission rates (1 SR).

**Table 3:** The results of the 17 included SRs for the patient-related characteristics of interest. The “overall” row for each characteristic shows the pooled results (based on the direction of effect) for each outcome measure for total hip arthroplasty, total knee arthroplasty and mixed total hip/knee arthroplasty separately. The up (↑) arrows denote statistical significance (higher odds or risks) in cases versus controls and the constant arrow ( ↔) denotes no statistical significance. The number in parentheses next to the direction of the effect arrow in the “overall” rows denote the number of pooled SRs of that result

Characteristic	SR	THA/TKA	Overall complications	Overall surgical complications	Overall medical complications	PJI	Aseptic loosening	THA dislocations	Revision	LOS	Re-admission	Mortality	Confidence in SR results
Smoking	Singh (2011) [1]	THA	↑ RR 1.24^HNR^	-	-	↔ RR 3.42^HNR^	↔ RR 1.25^HNR^	-	↔ RR 1.14^HNR^	-	-	↑ RR 1.63^HNR^	Critically low
	Teng et al. (2015) [17]	THA	-	-	-	↑ RR 3.71	↑ RR 3.05	↔ RR 1.27	↑ RR 2.58^H^	↔ MD 0.03	-	-	Critically low
	Kunutsor et al. (2016) [2]	THA/TKA	-	-	-	↑ RR 1.83^HNR^	-	-	-	-	-	-	Critically low
	Kong et al. (2017) [6]	THA/TKA	-	-	-	↔ OR 1.48	-	-	-	-	-	-	Critically low
		TKA	-	-	-	↔ OR 1.48	-	-	-	-	-	-	
	Bedard et al. (2019) [15]	THA/TKA	-	-	-	↑ OR 2.02^HNR^	-	-	-	-	-	-	Critically low
	Bojan et al. (2020) [16]	THA	-	-	-	↑ OR 1.81	-	-	↑ OR 2.02	-	-	-	Critically low
	Ren et al. (2021) [7]	THA	-	-	-	↔ RR 1.24	-	-	-	-	-	-	Critically low
	Resende et al. (2021) [14]	THA/TKA	-	-	-	↑ OR 1.82^H^	-	-	-	-	-	-	Critically low
	He et al. (2022) [18]	TKA	↑ RR 1.22^H^	↑ RR 1.71	↔ (pneumonia ↑ RR 1.45^H^)	↑ RR 2.05	-	-	↑ RR 1.31	-	-	↔ RR 1.83^H^	Low
	**Overall (number of pooled SRs)**	THA	**↑ (1)**	-	-	**CE (4)**	**CE (2)**	** ↔ (1)**	**↑ (2)**	** ↔ (1)**	-	**↑ (1)**	-
		TKA	**↑ (1)**	**↑ (1)**	** ↔ (pneumonia ↑) (1)**	**↑ (2)**	-	-	**↑ (1)**	-	-	** ↔ (1)**	-
		THA/TKA	-	-	-	**↑ (4)**	-	-	-	-	-	-	-
Alcohol excess	Kunutsor et al. (2016) [2]	THA/TKA	-	-	-	↔ RR 2.84^HNR^	-	-	-	-	-	-	Critically low
	Kong et al. (2017) [6]	THA/TKA	-	-	-	↑ OR 1.88	-	-	-	-	-	-	Critically low
	Ren et al. (2021) [7]	THA	-	-	-	↑ RR 1.69^H^	-	-	-	-	-	-	Critically low
	Resende et al. (2021) [14]	THA/TKA	-	-	-	↑ OR 2.95^H^	-	-	-	-	-	-	Critically low
	**Overall (number of pooled SRs)**	THA/	-	-	-	**↑ (1)**	-	-	-	-	-	-	-
		THA/TKA				**↑ (3)**							-
Rheumatoid Arthritis	Ravi et al. (2012) [19]	THA	-	-	-	-	-	↑ OR 2.16	↔ ^a^ OR 0.91	-	-	↔ OR 1.40^H^	Critically low
		TKA	-	-	-	-	-	-	↔ ^a^ OR 2.02^H^	-	-	↔ OR 0.86	
	Lee et al. (2017) [20]	THA	-	-	-	↑ OR 2.04	-	-	↔ OR 1.22^b^	-	-	-	Critically low
	Kong et al. (2017) [6]	THA	-	-	-	↑ OR 1.75	-	-	-	-	-	-	Critically low
		TKA	-	-	-	↑ OR 1.34							
		THA/TKA	-	-	-	↑ OR 1.57							
	Ren et al. (2021) [7]	THA	-	-	-	↑ RR 1.37	-	-	-	-	-	-	Critically low
	**Overall (number of pooled SRs)**	**THA**	**-**	**-**	**-**	**↑ (3)**	**-**	**↑ (1)**	** ↔ (2)**	**-**	**-**	** ↔ (1)**	**-**
		**TKA**	**-**	**-**	**-**	**↑ (1)**	**-**	**-**	** ↔ (1)**	**-**	**-**	** ↔ (1)**	**-**
		**THA/TKA**	**-**	**-**		**↑ (1)**	**-**	**-**	**-**	**-**	**-**	**-**	**-**
Hepatitis C	Wei et al. (2019) [23]	THA	↑ HR 1.25	-	-	↑ HR 2.69	-	-	↑ HR 2.21^H^	↑ HR 2.05^H^	-	-	Critically low
		TKA	↑ HR 1.55^H^	-	-	↔ HR 1.41	-	-	↔ HR 1.53	↑ HR 4.61^H^	-	-	
		THA/TKA	↑ HR 1.55^H^	-	-	↑ HR 2.04	-	-	↑ HR 2.51	↑ HR 0.88^H^	-	-	
	Cheng et al. (2022) [24]	THA	↑ OR 1.53^H^	↑ OR 1.72^H^	↑ OR 1.72^H^	↑ OR 1.71	-	-	↑ OR 1.59	-	-	-	Critically low
		TKA	↑ OR 1.67^H^	↑ OR 1.75^H^	↑ OR 1.86^H^	↑ OR 2.06	-	-	↑ OR 1.40	-	-	-	
		THA/TKA	↑ OR 1.53^H^	↑ OR 1.58^H^	↑ OR 1.72^H^	↑ OR 2.72	-	-	↑ OR 1.47	-		-	
	**Overall (number of pooled SRs)**	**THA**	**↑ (2)**	**↑ (1)**	**↑ (1)**	**↑ (2)**	-	-	**↑ (2)**	**↑ (1)**	-	-	-
		**TKA**	**↑ (2)**	**↑ (1)**	**↑ (1)**	**CE (2)**	-	-	**CE (2)**	**↑ (1)**	-	-	-
		**THA/TKA**	**↑ (2)**	**↑ (1)**	**↑ (1)**	**↑ (2)**	-	-	**↑ (2)**	**↑ (1)**	-	-	-
HIV	Dimitriou et al. (2017) [22]	THA/TKA	↑ RR 2.28^HNR^	-	-	↑ RR 2.28^HNR^	-	-	↔ ^HNR^	-	-	-	Critically low
	O’ Neill et al. (2019) [21]	THA/TKA	-	-	-	↑ RR 3.31	-	-	↑ RR 2.25	-	-	-	Critically low
	**Overall (number of pooled SRs)**	**THA/TKA**	**↑ (1)**	**-**	**-**	**↑ (2)**	**-**	**-**	**CE (2)**	-	-	-	-
Depression	Kim & Kim 2021 [25]	THA/TKA	**-**	**-**	**-**	**-**	**-**	**-**	**-**	**-**	↑ OR 1.27^H,90^	**-**	Critically low
		THA	**-**	**-**	**-**	**-**	**-**	**-**	**-**	**-**	↔ OR 1.51^H,90^	**-**	
		TKA	**-**	**-**	**-**	**-**	**-**	**-**	**-**	**-**	↑ OR 1.28^H,90^	**-**	
	**Overall (number of pooled SRs)**	**THA/TKA**	**-**	**-**	**-**	**-**	**-**	**-**	**-**	**-**	**↑ (1)**	**-**	**-**
		**THA**	**-**	**-**	**-**	**-**	**-**	**-**	**-**	**-**	↔ **(1)**	**-**	**-**
		**TKA**	**-**	**-**	**-**	**-**	**-**	**-**	**-**	**-**	**↑ (1)**	**-**	**-**
Solid organ transplant	Kim et al. (2022**)** [26]	**THA**	**-**	**-**	**-**	↔ OR 1.75^H^	↔ OR 0.77	↔ OR 1.58^H^	↔ OR 0.9^H^	**-**	↑ OR 1.65	↑ OR 2.02	Critically low
	**Overall (number of pooled SRs)**		**-**	**-**	**-**	** ↔ (1)**	** ↔ (1)**	** ↔ (1)**	** ↔ (1)**	**-**	**↑ (1)**	**↑ (1)**	**-**

*CE* Conflicting evidence, *HR* Hazard ratio, *LOS* Length of stay, *RR* Relative risk (risk ratio), *OR* Odds ratio, *PJI* Periprosthetic joint infection, *SR* Systematic review

^H^: significant statistical heterogeneity (I^2^ > 50%)

^HNR^: heterogeneity tests not reported

^90^: 90-day re-admission

^a^Similar incidence 6–10 years postop, however greater incidence up to 5 years and lower incidence for THA after 10 years (similar incidence for TKA)

^b^Revision for non-infective causes

## Discussion

To the best of our knowledge this is the first review summarising and appraising SRs that assess primary THA and TKA outcomes in patients with these specific pre-defined patient-related characteristics. The most striking finding of our study was the poor quality of the included SRs as shown from the application of the AMSTAR-2 tool, which is the most widely recognised critical appraisal tool for SRs that include randomised or non-randomised studies of healthcare interventions. Although the focus of these SRs was assessment of specific patient-related characteristics rather than the intervention itself, which was the same in “cases” and “controls”, the same principles should apply, and the same methodological and reporting criteria should be fulfilled. As an example, only two of the 17 included SRs assessed and reported the strength of evidence of their results and only one of the two used a recognised method [14, 19]. Another interesting finding was the lack of assessment of patient-reported outcome measures (PROMs); we argue that PROMs such as quality of life, satisfaction, pain and function should be regarded as equally important as peri-operative complications as they should also form part of the pre-operative decision-making process.

Tobacco use, which was the characteristic assessed by the largest number of included SRs, was generally found to be a risk factor for worse outcomes in both THA and TKA. PJI was the outcome most assessed by the included SRs and even though the evidence was conflicting for THA alone, the evidence for mixed THA/TKA data and that of TKA alone demonstrated higher PJI rates in smokers with RRs as high as 4.55. Singh (2011) reported a number needed to harm of 34 for any postoperative complication and mortality associated with tobacco use [1]. An early randomised controlled trial by Moller et al. (2002) demonstrated clear benefits of smoking cessation before a TKA and THA with regard to wound complications, cardiovascular complications and LOS [27]. Additionally, a Cochrane SR of 13 randomised controlled trials by Thomsen et al. (2014) also demonstrated that pre-operative smoking cessation was effective at reducing postoperative complications in surgery in general, including non-orthopaedic surgery [28]. Interestingly, former use of tobacco was generally associated with higher odds for complications compared to never use; however, these were lower than current use [1, 15, 17].

Alcohol excess as a possible risk factor for peri-operative THA and TKA complications was only assessed by large SRs among several other patient-related characteristics for PJI only. ORs for PJI compared to controls in two SRs ranged from 1.88–2.95 and RR from 1.69–2.84 in another 2 SRs [2, 6, 14, 17]. Outcomes other than PJI have not been investigated within SRs to the best of our knowledge. A large retrospective study by Best et al. (2015) demonstrated that alcohol misuse was independently associated with higher odds of in hospital complications, overall surgical and medical complications and LOS after elective THA and TKA [28]. Those who misused alcohol were found to be 15 times more likely to develop any acute postoperative infection compared to controls [29]. These findings are in agreement with those of two recent retrospective studies, one in THA and the other in TKA, both showing significantly higher LOS, increased odds of medical complications (including venous thromboembolism), two-year implant-related complications and healthcare costs [30, 31]. Low to moderate alcohol consumption, on the other hand, was associated with lower rates of 90-day and one-year mortality as well as 30-day cardiovascular disease after THA or TKA compared to abstainers [32].

RA may be associated with an increased incidence of PJI and dislocation in THA based on the existing literature. Mortality is likely similar to OA patients after THA and TKA. A SR by Taylor-Williams et al. (2020) on THA only looked at complications and how they evolved from the 1980’s to the 2010’s [33]. Overall complications decreased in the 2010’s (5.3%) compared to the two preceding decades (9.9% in 1990’s and 12.7% in 2000’s) and so did revision rates (6.2%, vs. 8.5% in 2000’s and 8.1% in 1990’s). Infection rates remained constant since the 1980’s with a mean incidence of 2.6% (2–6 times higher than the general population). Dislocation rates increased from 0.4% in the 2000’s to 1.5% in the 2010’s and aseptic loosening rates also increased from 2.8% in the 1990’s to 3.8% in the subsequent decades [33]. These chronological changes reflect a complex interplay between advances in medical and surgical care and wider use of immunosuppressive and other medications for the management of RA. It remains unclear whether this increased risk of PJI in RA patients after THA is related to the disease itself or the medications that these patients are frequently treated with.

Infection with HIV and HCV likely increases the risk for complications after THA and TKA based on our findings. The benefits of pre-operative treatment with antivirals in HCV patients were assessed by Cheng et al. (2022) with their SR of eight studies [34]. Among patients who had TKA and THA, compared to those who did not receive antiviral therapy pre-operatively, those who did had significantly lower incidences of overall complications in THA but not TKA, surgical complications for both THA and TKA and PJIs for both THA and TKA. Additionally, a multicentre retrospective study by Novikov et al. (2019) showed that, compared to those with HCV that had a detectable viral load, undetectable viral loads were associated with a significantly lower incidence of complications including infection, postoperative blood loss and revision [35]. Conversely, anti-retroviral treatment for HIV prior to THA did not offer significant benefits with regard to peri-operative complications according to the retrospective study by Sax et al. (2021) as the differences did not reach statistical significance; generally, however, HIV patients (treated and untreated) had very similar odds for complications compared to patients without HIV [36]. Indeed, Falakassa et al. (2014) found that HIV patients on highly active anti-retroviral therapy with an undetectable viral load and CD4 count > 200 were at similar risk of PJI as the general population [37].

The single SR that we identified on mental health patients only assessed pre-operative depression as a risk factor for higher odds for re-admission after THA or TKA. The authors found increased odds of 90-day re-admission after TKA and mixed THA/TKA data but not THA alone compared to controls [25]. The authors did not specify reasons for the higher incidence of re-admission after TKA in those with depression but retrospective studies demonstrated increased odds of haematoma, postoperative infection, postoperative anaemia, other medical complications as well as implant-related complications among others in those with depression compared to controls [38, 39]. Treatment of depression was not considered or adjusted for, however a retrospective study by Halawi et al. (2020) demonstrated that treatment of depression was not associated with significant benefits in PROMs [40]. Greene et al. (2016) showed in their retrospective multicentre study that a pre-operative diagnosis of anxiety/depression (use of anti-depressants) was associated with worse PROMs compared to controls one year after THA [41]. Psychiatric disorders (bipolar, depression and schizophrenia) were also found to be a risk factor for increased surgical and medical complication rates after both TKA and THA, including PJI ORs of 2.17 and 2.26 respectively [42, 43].

Finally, in the included SR, although primary THA in those with solid organ transplants was associated with similar incidences of surgical complications compared to controls, there was a significantly greater incidence of cardiac complications, pneumonia and acute kidney injury [26]. Transfusion rates and 90-day re-admission rates were also found to be higher in those with solid organ transplants. A recent SR [44] assessing outcomes of THA and TKA in liver transplant recipients was not included in our study; even though quantitative data were presented by the authors, these are purely ORs based on mean percentage incidences of cases versus controls without forest plots and the relevant appropriate methodology. The authors found that liver transplants were associated with significantly worse outcomes compared to controls for most outcomes (surgical and medical) after a THA and TKA. They report that there was significant heterogeneity in demographic information and the outcomes in the included studies.

A few SRs investigating haemophilia and prior intra-articular knee fracture as possible risk factors for higher complication rates after total joint arthroplasty had to be excluded as they were either purely narrative and did not provide any pooled statistics or did not include comparisons with controls. The SR by Pander et al. (2021) reported similar PROMs but higher overall complication and re-operation rates in TKA patients with a previous tibial plateau fracture compared to matched primary OA controls and the SR by Tapper et al. (2021) found that overall complication and revision rates were lower in those who had TKA as primary treatment for their tibial plateau fracture compared to those who had a delayed TKA but higher compared to elective primary TKA [45, 46]. SRs on TKA in haemophilia patients reported significant postoperative improvements in PROMs and functional outcomes with the highest complication being haemarthroses in 7%–8% of patients, approximately one-third of whom needed surgical evacuation of the haematoma [47, 48]. Implant survivorship was 84% at 15 years in one SR and 93.7% at 6.3 years in another SR [47, 48]. Complications after THA in haemophilia patients were found to be comparable to the general population in another SR [49]. Perioperative factor replacement in haemophilia is widely accepted to be a necessary strategy to minimise complications [50].

Our review has limitations. First, the level of evidence of the studies within the included SRs being observational, mostly retrospective, is low as the nature of the topic precludes the conduct of randomised studies and additionally, most of them did not adjust for confounders. Second, the critical appraisal tool of all included SRs revealed important methodological and reporting flaws which makes confidence in their results very low. Finally, we could not perform quantitative pooling of results as there was an overlap of studies within the pooled SRs and the generalisability of most findings is questionable as the vast majority of studies were conducted in a single country (USA). As a result, the present study's findings should be interpreted and conveyed to patients with caution as the poor quality of the available evidence precludes definitive conclusions, predominantly due to the flaws of the included studies.

Further high-quality research is necessary on patient-related characteristics to provide results with higher strength of evidence. Importantly, PROMs should be assessed more in future research as they should be as important of a consideration as complications for decision-making prior to THA and TKA.

## Conclusion

We found that smoking, excess alcohol consumption, RA, and infection with HIV and HCV were associated with a higher incidence of PJI in one or both of THA and TKA or mixed THA/TKA data. Our review provides a synopsis of the highest available quality evidence on peri-operative outcomes of THA and TKA in patients with special characteristics, which can be used by healthcare professionals when consulting patients during the decision-making and informed consent processes. The included SRs in our review were of poor quality, therefore the results should generally be interpreted with caution as the strength of the available evidence is not high enough for definitive conclusions.

## Data Availability

All data is available upon request.
